# Early life predictors of midlife allostatic load: A prospective cohort study

**DOI:** 10.1371/journal.pone.0202395

**Published:** 2018-08-16

**Authors:** Dinne Skjærlund Christensen, Trine Flensborg-Madsen, Ellen Garde, Åse Marie Hansen, Jolene Masters Pedersen, Erik Lykke Mortensen

**Affiliations:** 1 Section of Environmental Health, Department of Public Health, University of Copenhagen, Copenhagen, Denmark; 2 Center for Healthy Aging, University of Copenhagen, Copenhagen, Denmark; 3 Danish Research Centre for Magnetic Resonance, Centre for Functional and Diagnostic Imaging and Research, Copenhagen University Hospital Hvidovre, Hvidovre, Denmark; 4 Section of Social Medicine, Department of Public Health, University of Copenhagen, Copenhagen, Denmark; 5 National Research Centre for the Working Environment, Copenhagen, Denmark; University of Colorado Denver, UNITED STATES

## Abstract

**Background:**

Allostatic load has been suggested as a pathway through which experiences become biologically embedded to influence health. Research on childhood predictors of allostatic load has focused on socioeconomic and psychosocial exposures, while few studies include prospective measures of biomedical exposures. Further, findings on sex differences in the association of childhood predictors with various health outcomes related to allostatic load are ambiguous.

**Aims:**

To examine the influence of early life biomedical and social factors in the first year of life on midlife allostatic load, assessing potential sex differences.

**Methods:**

This prospective cohort study includes early life information collected at birth and a one year examination for 1,648 members of the Copenhagen Perinatal Cohort who also participated in the Copenhagen Aging and Midlife Biobank study (aged 49–52 years, 56% women). Allostatic load based on 14 biomarkers was selected as a measure of midlife health status. Early life factors were categorized as predominantly biomedical or social, and their associations with midlife allostatic load were examined in domain-specific and combined sex-stratified multiple regression models.

**Results:**

The biomedical factors model explained 6.6% of the variance in midlife allostatic load in men and 6.7% in women, while the social model explained 4.1% of the variance in men and 7.3% in women. For both sexes, parental socioeconomic position at one year and maternal BMI significantly predicted midlife allostatic load in a model containing all early life factors. For women, additional significant predictors were complications at birth, birth weight and not living with parents at one year.

**Conclusion:**

The results confirm an association of lower childhood socioeconomic position with higher adult allostatic load while demonstrating the importance of other prenatal and early life exposures and highlighting potential sex differences.

## Introduction

While the association between childhood socioeconomic position (SEP) and later health is well-established [1–4], the causal mechanisms by which childhood SEP ‘gets under the skin’ remain unclear [5–7]. One hypothesized pathway is *allostasis*: The automatic, neuroendocrine, immune and behavioral process by which we seek to adapt to various stressors in our environment throughout life [8]. Over time, this process of adaptation may result in physiological dysregulation, and potentially lead to disease [9]. Allostatic load (AL) is a measure of physiological dysregulation hypothesized to reflect such ‘wear and tear’ across the lifespan [10]. The definition and operationalization of AL make it useful for analyses of the link between childhood SEP and later health in several ways: First, AL reflects dysregulation at a preclinical level, enabling insight to the antecedent mechanisms by which early life factors are associated with later disease and morbidity [11]. Secondly, AL is multidimensional, subsuming biomarkers across several interdependent biological systems. Combined, these biomarkers predict disease and mortality better than individual markers [12], and AL has been shown to partially mediate the SEP-mortality association in older adults [13]. Finally, although AL is conceptualized within a life course framework, major emphasis is placed on how the earliest stages of life shape subsequent perception of and response to potential stressors and environmental challenges [9].

Studies have confirmed associations between low childhood SEP and potentially related psychosocial factors such as abuse and maltreatment, parental death or divorce, and social isolation with increased levels of later AL [8,11,14–16]. However, despite the emphasis within the AL framework on the earliest life stages, few studies include pre-, peri-, and early postnatal factors, and most studies rely on retrospective self-reports of exposure [11,16–19]. Furthermore, the majority of studies focus on socioeconomic and/or psychosocial factors [15–17,20], despite the well-established association of low SEP with exposures of an inherently biological or more directly health-related nature, e.g. various prenatal risk factors (such as maternal smoking and certain infections during pregnancy) and adverse pregnancy outcomes (such as preterm birth and low birth weight) [21]. Considering the relation of these early life biomedical factors with adult health outcomes [22–24], it is likely that such factors play a substantial role in the link between childhood SEP and adult AL. Focusing on such factors is thus essential to improve our understanding of how childhood SEP comes to affect adult AL. Finally, among the few studies examining sex differences in associations of childhood factors with AL, results are inconclusive [19,25–27]. As sex differences have been found in studies of the association of childhood SEP with health outcomes related to AL [28–33], this issue warrants further research.

Using prospective data from a Danish birth cohort, the aims of the present study were to evaluate the extent to which early life biomedical and social factors are associated with midlife AL and to examine potential sex differences in these associations.

## Materials and methods

### Study population

The Copenhagen Perinatal Cohort (CPC) consists of 9,125 individuals born to 8,949 mothers at the Copenhagen University Hospital during October 1959–December 1961, with 8,400 children surviving the first month. The mothers were predominantly residents of the Copenhagen area, although some were referred due to single mother status or obstetrical complications. Information on demographic, socioeconomic, pre-, peri-, and postnatal medical factors was collected in personal interviews during pregnancy, at delivery, and at a 1-year examination; this data collection is described in detail elsewhere [34].

During 2009–2011, the Copenhagen Aging and Midlife Biobank (CAMB) was established as a follow-up of three cohorts: the Danish Longitudinal Study on Work, Unemployment, and Health consisting of a random sample of Danish men and women born in 1949 and 1959 [35], the Metropolit Cohort consisting of men born in Copenhagen in 1953 [36], and the CPC. Due to practical and financial reasons, cohort members residing in western Denmark were excluded. In total, 5,282 members of the CPC were invited to participate [37]. The follow-up consisted of a postal questionnaire and an extensive health examination including non-fasting blood samples for 1,718 members of the CPC, resulting in a 32.5% participation rate. Participants were between the ages of 49 and 52 years [38]. Upon enrollment in the CAMB study, all participants provided informed consent. The study protocol was approved by the Danish National Committee on Health Research Ethics (No. H-A-2008-126) and the Danish Data Protection Agency (No. 2008-41-2938).

Among the 1,718 participants with blood samples, 53 twins were excluded to ensure statistical independence of the data and comparability of perinatal factors such as birth weight. Of the remaining 1,665 participants, AL was not calculated for 17 participants for whom less than seven biomarkers were available. The final study sample consisted of 1,648 participants.

### Allostatic load

The AL index score used in the current study was based on 14 biomarkers collected at midlife (mean age 49.9 years) representing the cardiovascular (systolic and diastolic blood pressure, averaged across four measurements), immune (interleukin 6 (IL-6), tumor necrosis factor α (TNF-α), high sensitivity C-reactive protein (hsCRP)) and metabolic system (low density lipoprotein (LDL), high density lipoprotein (HDL), total cholesterol, body mass index (BMI), waist/hip ratio (WHR), blood glucose, triglycerides, HbA_1c_, percent body fat). Data collection and blood sample analyses are described in detail by Hansen *et al*. [39]. The AL score was calculated using the traditional method of summing the number of AL markers falling in the high-risk quartile [40]. The high-risk quartile was defined as the within-sample sex-specific 75^th^ percentile, except in the case of HDL, for which high risk was defined as below the 25^th^ percentile. Cut-points are provided in S1 Table. Biomarkers were then dichotomized reflecting whether or not their values were in the high-risk quartile, and summed for an index ranging from 0–14. For six participants, values of IL-6 were below the level of detection, in which case values were imputed from a uniform distribution between zero and a limit of detection. Information on all 14 biomarkers was available for 97.8% of participants. For participants with less than all 14 but at least half (seven or more) of the included biomarkers available (n = 18), the AL score was calculated as the mean of the available biomarkers, multiplied by the total number of biomarkers (14).

### Early life predictors

Based on relevant epidemiological findings and theoretical considerations, potential predictors were selected among information on early life factors collected at birth and the 1-year examination and divided into predominantly biomedical or social factors.

The following factors were selected as potential biomedical predictors: Maternal smoking in the final trimester of pregnancy (no, yes), gestational age (weeks), presence of one or more birth complications, e.g. mechanical hindrance, weak labor, precipitous birth or ‘other’ (no, yes), hospital stay within the first year (no, yes), birth weight (grams), maternal BMI (weight in kg/(height in m)^2^), duration of breastfeeding (months), and maternal age at birth (years).

The following factors were selected as potential social predictors: Attitude toward the pregnancy (child wanted vs. child unwanted), marital status of the mother at conception (married vs. unmarried), change in maternal marital status from conception to one year (no, yes), whether the child was living with parent(s) at one year (yes, no), and parental SEP. Information on parental SEP was collected at the 1-year examination and was based on points from 0–5 for four factors in accordance with the social grouping of the Centre International de l’Enfance [41]: 1) education (0 representing basic schooling and 5 representing attainment of a university degree) and 2) occupation of breadwinner (0 representing labourers and 5 representing business or professional occupation), 3) type of income of breadwinner (0 representing public relief and 5 representing own business or capital), and 4) quality of living accommodation (according to its size and number of persons pr. room). In relation to the initial computerization of the data in the 1970s, points on the resulting 0–20 point scale were originally transformed to a 1–9 point scale to save space on punch cards, with higher points indicating increasing SEP. For the present sample, group 8 and 9 were collapsed due to low frequencies in the upper group and the resulting 1–8 point scale was analyzed as a linear continuous variable.

### Statistical analyses

Independent samples *t*-tests and chi-square tests were conducted to examine gender differences in all variables. For early life predictors, the missing data rate ranged from 0% (sex and whether the child was living with parents at one year) to 16.9% (gestational age). The largest amount of missing data was found for gestational age (16.9%), duration of breastfeeding (14.4%) and parental SEP (15.8%). This is likely due to the fact that information on duration of breastfeeding and parental SEP was collected at the 1-year examination, for which there was some attrition, and for 20% of the full CPC sample gestational age could not be established with certainty [42]. Because information on all variables was collected by medical doctors rather than by self-report, we consider it reasonable to assume information to be missing at random. Due to sex differences in associations of parental SEP with health outcomes related to AL [28–33], and because formal tests of interaction with sex were significant (*p* < .05) or marginally significant (*p* < .10) for four factors (maternal smoking in the final trimester (*p* = .058), complications at birth (*p* = .072), maternal BMI (*p* = .011) and not living with parents at one year (*p* = .032), all analyses were stratified by sex.

For the main statistical analyses, associations of the biomedical and social factors with AL were first examined separately in domain-specific linear regression models 1 and 2. Further, the information on biomedical and social predictors available from the birth cohort data enabled a test of whether the well-established associations of social factors with AL persist when accounting for early life biomedical exposures, which often co-occur with social exposures [21]. Model 3 thus included both biomedical and social predictors. All models were adjusted for the following covariates: age at follow up (years), time of day of blood draw, and whether or not participants were fasting within two hours of blood draw (fasting, not fasting). The amount of variance explained by individual predictors and models were estimated by increment *R*^2^ (semipartial *R*^2^) and model *R*^2^, respectively. To handle missing data and increase comparability between models, we used Stata’s structural equation modeling procedure with full information maximum likelihood (FIML) estimation, which uses all available information rather than listwise deletion [43]. Sensitivity analyses using a complete case sample were also performed.

Two additional sensitivity analyses were conducted: first, though medical treatment for conditions related to AL (e.g. hypertension) may influence biomarkers levels, unfortunately information on medical treatment status was not available. However, to address the fact that certain biomarkers may be influenced by recent infections and current morbidity, a sensitivity analysis was performed adjusting for self-reported systemic infections (fever, cold, flu, pneumonia, digestive or urinary tract infection, or other infections) within the past 3 weeks (n = 438), hypertension (n = 222) or diabetes (n = 24). Second, all analyses were performed using an alternative AL index based on clinical rather than sample-based cut-points where available. Clinical cut-points were derived from established criteria [44,45] and are displayed in S1 Table. All analyses were conducted in Stata 14 (StataCorp. 2015) using robust standard error estimation.

Collinearity was evaluated by variance inflation factors (VIF) and did not indicate any problems (VIF for all variables below 5 in all models [46]). Normality of residuals was evaluated graphically and revealed no substantial deviations. Linearity of regression was evaluated by testing the significance of a quadratic component for all continuous predictors, none of which were significant.

## Results

Table 1 displays proportions or means and standard deviations for age, midlife AL score, and all early life factors. The final sample consisted of 56% women. Men and women did not significantly differ in mean AL scores or early life factors aside from birth weight. Approximately half of the children were classified as ‘unwanted’, reflecting that around 30% of the mothers were unmarried at conception (single mother status was one of the criteria for referral to the Copenhagen University Hospital).

**Table 1:** Sex-stratified descriptive statistics of early life biomedical and social factors including test for sex differences.

	n	Men	n	Women	*p*^a^
Age at follow-up (mean[min; max])	726	49.9 (49;52)	922	49.9 (49;52)	.57
Allostatic load score (mean[SD])	726	3.47 (2.66)	922	3.42 (2.74)	.72
Biomedical factors					
Maternal smoking in the final trimester (N[%])	710	328 (46.2)	899	346 (48.5)	.36
Gestational age, weeks (mean[SD])	591	39.07 (2.6)	778	39.2 (2.3)	.24
Complications at birth (N[%])	711	66 (9.3)	904	71 (7.9)	.31
Hospital stay in the first year (N[%])	712	137 (19.2)	892	146 (16.4)	.13
Birth weight, grams (mean[SD])	718	3326 (587.5)	906	3178 (540.5)	< .001
Maternal BMI (mean[SD])	659	21.7 (2.85)	839	21.6 (2.81)	.29
Duration of breastfeeding, months (mean[SD])	625	3.3 (2.76)	785	3.3 (2.70)	.99
Maternal age at birth, years (mean[SD])	726	26.2 (6.66)	922	25.9 (6.71)	.47
Social factors					
Attitude toward the pregnancy, unwanted (N[%])	704	351 (49.7)	889	390 (53.1)	.20
Marital status of mother at conception, single (N[%])	716	211 (29.5)	910	303 (33.3)	.099
Change in marital status, conception to 1 year (N[%])	654	76 (11.6)	821	108 (13.2)	.38
Not living with parents at 1 year (N[%])	726	34 (4.7)	922	33 (3.6)	.26
Parental SEP at 1 year, 1–8, low to high (mean[SD])	610	4.38 (1.88)	777	4.33 (1.89)	.62

^a^ *t*-test or *x*^2^ test for sex differences

Zero-order inter-correlations among study variables are presented in S2 Table. Maternal smoking, maternal BMI, marital status of mother at conception and change in marital status within the first year were positively associated with AL (all *p* < .05). Higher birth weight, longer duration of breastfeeding, higher maternal age at birth and higher parental SEP at one year were associated with lower AL. Further, significant associations were observed among parental SEP and most of the additional factors, except gestational age, complications at birth and not living with parents at one year. Table 2 displays the sex-stratified results of domain-specific models 1 and 2.

**Table 2:** Domain-specific linear regression models predicting midlife allostatic load from early life biomedical and social factors^a^.

	Men (N = 726)		Women (N = 922)	
	β [95% CI]	% Incr. *R*^*2*^	β [95% CI]	% Incr. *R*^*2*^
**Model 1.** Biomedical factors	Model *R*^2^ = 0.0658		Model *R*^2^ = 0.0670	
Maternal smoking in the final trimester	0.02 [-0.383; 0.418]	0.01%	0.43 [0.054; 0.814]*	0.50%
Gestational age, weeks	0.07 [-0.031; 0.166]	0.27%	0.08 [-0.028; 0.190]	0.28%
Complications at birth	-0.18 [-0.830; 0.474]	0.03%	0.78 [0.137; 1.413]*	0.58%
Hospital stay in the first year	0.30 [-0.225; 0.833]	0.16%	-0.12 [-0.676; 0.438]	0.02%
Birth weight, grams	-0.0004 [-0.001; 0.00004]^†^	0.45%	-0.001 [-0.001; -0.0001]*	0.64%
Maternal BMI	0.21 [0.135; 0.285]***	4.57%	0.10 [0.030 0.168]**	0.99%
Duration of breastfeeding, months	-0.02 [-0.098; 0.059]	0.04%	-0.04 [-0.106 0.036]	0.12%
Maternal age at birth	-0.03 [-0.062; -0.003]*	0.71%	-0.04 [-0.068; -0.013]**	0.85%
**Model 2.** Social factors	Model *R*^2^ = 0.0405		Model *R*^2^ = 0.0733	
Attitude towards the pregnancy, unwanted	-0.34 [-0.794; 0.112]	0.18%	-0.19 [-0.601; 0.218]	0.00%
Marital status of mother at conception, single	0.29 [-0.288; 0.861]	0.03%	0.14 [-0.369; 0.648]	0.00%
Change in marital status, conception to 1 year	-0.07 [-0.801; 0.662]	0.00%	0.45 [-0.189; 1.098]	0.49%
Not living with parents at 1 year	-0.03 [-0.982; 0.922]	0.03%	1.34 [0.385; 2.298]**	0.59%
Parental SEP at 1 year	-0.25 [-0.371; -0.133]***	2.59%	-0.28 [-0.379; -0.171]***	2.78%

*Note*. % Increment *R*^2^ reflects the variable-unique explained variance.

^a^Unstandardized coefficients based on Full Information Maximum Likelihood (FIML) estimation, adjusted for age at follow-up, time of blood draw, and fasting status within two hours of blood draw.

^†^ *p* < .10

* *p <* .05

** *p <* .01

*** *p <* .001

In men, higher maternal BMI was significantly associated with higher AL at midlife, and higher maternal age at birth was significantly associated with lower AL in the biomedical model (model 1). Parental SEP at one year was the only significant predictor in the social model (model 2), with lower levels of SEP related to higher AL scores. In women, several biomedical factors significantly predicted AL: maternal smoking in the final trimester, complications at birth and higher maternal BMI were associated with higher midlife AL, while birth weight and maternal age were inversely associated with AL. In the social model, not living with parents at the age of one year and parental SEP at one year were significant predictors of AL, indicating higher levels of AL among women who did not live with their parents at age one year. In both sexes, maternal BMI explained the largest amount of variance in the biomedical model while parental SEP at one year explained the largest amount of variance in the social model.

Table 3 shows the results of model 3, combining the biomedical and social predictors.

**Table 3:** Linear regression model predicting midlife allostatic load from early life biomedical and social factors, combined^a^.

	Men (N = 726)		Women (N = 922)	
	β [95% CI]	% Incr. *R*^*2*^	β [95% CI]	% Incr. *R*^*2*^
**Model 3**. Combined model	Model *R*^2^ = 0.0844		Model *R*^2^ = 0.0950	
Maternal smoking in the final trimester	-0.02 [-0.417; 0.378]	0.01%	0.33 [-0.051; 0.716]^†^	0.27%
Gestational age, weeks	0.06 [-0.041; 0.157]	0.18%	0.08 [-0.032; 0.184]	0.23%
Complications at birth	-0.20 [-0.844; 0.437]	0.04%	0.83 [0.201; 1.456]**	0.65%
Hospital stay in the first year	0.20 [-0.321; 0.718]	0.04%	-0.16 [-0.710; 0.398]	0.04%
Birth weight, grams	-0.0003 [-0.001; 0.0001]	0.36%	-0.001 [-0.001; -0.00004]*	0.53%
Maternal BMI	0.19 [0.113; 0.268]***	3.63%	0.08 [0.014; 0.152]*	0.58%
Duration of breastfeeding, months	-0.01 [-0.091; 0.068]	0.01%	-0.01 [-0.078; 0.061]	0.00%
Maternal age at birth	-0.01 [-0.044; 0.023]	0.41%	-0.01 [-0.043; 0.021]	0.00%
Attitude towards the pregnancy, unwanted	-0.47 [-0.910; -0.023]*	0.45%	-0.19 [-0.584; 0.214]	0.00%
Marital status of mother at conception, single	0.33 [-0.244; 0.912]	0.00%	0.03 [-0.484; 0.546]	0.00%
Change in marital status, conception to 1 year	-0.01 [-0.731; 0.721]	0.00%	0.58 [-0.068; 1.219]^†^	0.38%
Not living with parents at 1 year	-0.10 [-1.056; 0.848]	0.02%	1.12 [0.131; 2.116]*	0.41%
Parental SEP at 1 year	-0.20 [-0.320; -0.070]**	1.28%	-0.23 [-0.335; -0.115]***	1.73%

*Note*. % Increment *R*^2^ reflects the variable-unique explained variance.

^a^Unstandardized coefficients based on Full Information Maximum Likelihood (FIML) estimation, adjusted for age at follow-up, time of blood draw, and fasting status within two hours of blood draw.

^†^ *p* < .10

* *p <* .05

** *p <* .01

*** *p <* .001

When combining the biomedical and social factors in model 3, maternal age at birth was reduced to non-significance in both sexes. In men, maternal BMI and parental SEP remained significantly associated with AL, with maternal BMI displaying the largest squared semipartial correlation (increment *R*^2^) with AL (3.6%). Additionally, the effect of attitude toward the pregnancy was strengthened, indicating lower levels of AL among those for whom the mother characterized the pregnancy as unwanted (β = -0.47, *p* = .039). In women, all variables except maternal age at birth remained significantly or marginally significantly associated with midlife AL in the combined model; while the estimates for maternal smoking, not living with parents at one year and parental SEP were slightly attenuated, estimates for complications at birth and change in marital status were slightly strengthened. The largest increment *R*^2^ was found for parental SEP (1.7%). In men, explained variance (model *R*^2^) was 6.6% for the biomedical model and 4.1% for the social model. The combined model explained approximately 8.4% of the variance in midlife AL. In women, explained variance was 6.7% for the biomedical model and 7.3% for the social model. The combined model explained 9.5% of the variance in midlife AL for women.

In sensitivity analyses of models 1–3 based on complete cases, results were generally consistent with those based on FIML estimation in men, though in model 1 the effect of maternal age at birth was no longer significant (β = -0.02, *p* = .28). In women, some parameter estimates were attenuated to nonsignificance (complications at birth and birth weight in model 1 and 3), while others were strengthened: the estimate for not living with parents at one year was strengthened though no longer significant in model 2 (β = 2.43, *p* = .094) while strengthened and significant in model 3 (β = 4.28, *p* < .001), in which the effect of maternal smoking was also strengthened (β = 0.71, *p* = .005). In a second sensitivity analysis, adjusting for recent infections or current morbidity showed no substantial changes in results, though in model 3 the effect of attitude towards the pregnancy was attenuated to marginal significance in men (β = -0.38, *p* = .088), as was the effect of birth weight in women (β = -0.0004, *p* = .068). Finally, using an AL index based on clinical rather than sample-based cut-points resulted in slightly higher mean AL scores (4.33 in men and 3.54 in women). Though the overall results were unchanged, in model 1 the effect of maternal smoking was attenuated in women (β = 0.31, *p* = .062), maternal age at birth was attenuated to nonsignificance for both sexes in model 2, and attitude towards the pregnancy was no longer significant in men in model 3 (β = -0.28, *p* = .19).

## Discussion

### Summary of main findings

This study aimed to examine the influence of early life biomedical and social factors on midlife allostatic load, assessing potential sex differences. In the combined model, parental socioeconomic position at one year was a significant predictor of midlife AL for both sexes; lower parental SEP was associated with higher levels of AL. Additionally, maternal BMI was a significant positive predictor for both sexes, and, in women only, complications at birth, birth weight and not living with parents at one year were significantly associated with midlife AL.

### Previous research

While a number of studies have shown associations between adverse childhood experiences and later AL, very few studies have examined the prospective association of prenatal and early life factors with adult AL. Two studies on the 1958 British birth cohort recently examined the link between adverse childhood experiences and AL at age 44, adjusting for early life factors similar to those in the present study, e.g. birth weight, maternal smoking, maternal BMI and maternal age at birth [26,27]. To our knowledge, these are the only studies to examine sex differences in the prospective association of early life factors with midlife AL, though formal tests of interaction were not performed. Independent effects of paternal occupation at birth, maternal BMI and birth weight for both sexes, and additionally of childhood pathologies in men and maternal education [26] and maternal age at birth in women [27] persisted after including adult health behaviors, educational level, depression, marital status and social class in a path analysis. The present findings confirmed the association of parental SEP and maternal BMI with midlife AL for both women and men [26], although tests of interaction showed the latter association to be significantly stronger in men. The association of birth weight with AL was replicated in the present findings for women only. The present study found no association of hospitalization within the first year with AL, and contrary to findings in the British birth cohort, maternal smoking was significantly associated with AL in women.

Several studies have examined sex differences in associations of early life factors with outcomes related to AL. For example, the association of childhood SEP with later metabolic risk has been found to be stronger in women than in men [28–30,33]. Previous studies have also suggested sex differences in the influence of low birth weight on low-density lipoprotein and risk of cardiovascular disease [31,47,48], but formal tests of interaction did not support sex differences in the association of birth weight with midlife AL in the present study. Sex differences have also been indicated for the effects of later adversity on AL. A cohort study on the association of life-course adversity with AL at age 43 showed social adversity in adolescence to predict AL in women only, while adversity in young adulthood significantly predicted AL in men, suggesting that sex differences might persist for later exposures [49].

### Interpretation of findings

The field of life course epidemiology offers several possible interpretations of the observed associations and sex differences [50]. One is that the early life exposures included in this study exert independent, latent effects on midlife AL [6]. Supporting this interpretation, several studies have found the effects of childhood SEP and other factors such as maternal BMI and birth weight to remain after including potentially mediating effects such as adult SEP and stress exposure on the association with AL [17,26,27]. As the sex differences observed in the present study cannot be attributed to factors such as differences in the tendency to experience or report adverse events, or sex differences in exposure to early life factors (anthropometric factors aside), the observed sex differences would indicate sex-differential vulnerability to some prenatal and early life exposures. This has previously been found in relation to the effects of maternal smoking on birth weight [51]. Because the included data are primarily related to the mother and child, such vulnerability could also arise through sex-specific hereditary processes, as AL and some of its specific components (e.g. BMI) have been shown to have heritable predispositions [52,53].

Another interpretation would be that the observed early life exposures are precursors to a life-course trajectory or pathway of exposures, the effects of which can either add to, potentiate or offset those of earlier exposures [54]. Traditionally, AL is posited to reflect the physiological consequences of long-term stress exposure, consistent with such a pathway hypothesis [55]. Prospective findings of a compensatory effect of positive social relationships on the effect of economic adversity on AL support this interpretation [15], along with more recent findings that upward social mobility can mitigate the negative effects of low childhood SEP on inflammatory and metabolic markers [56]. Within this perspective, apparent sex differences in the effects of early life exposures are driven by sex differences in later exposures. For example, girls have previously been found to be more compliant with mothers and more likely to internalize maternal rules, relative to boys [57], suggesting that they are more influenced by their mother’s lifestyle and health-related behavior. Cohort effects might strengthen this mechanism, as the relatively gender-specific upbringing which was dominant at the time these participants were born likely meant that daughters spent more time with their mothers, allowing for greater similarities in behavioral factors related to AL to manifest. Alternatively, sex differences in health-related risks and behaviors [58] could cause the effects of some early life factors to be overshadowed by health-related factors in adulthood in men more so than in women, making early life exposures appear more significant for women. Finally, sex differences could arise through a sex-differential influence of early exposures on later exposures, creating more stable pathways of exposure for women. In line with the health selection hypothesis, poor childhood health has been found to be associated with lower adult SEP for both sexes [59]. It has been suggested that men and women have different opportunities to counteract such negative effects of early childhood factors through education or upward social mobility, resulting in less malleable trajectories for women [30]. However, within the present sample the correlation between parental SEP and offspring years of education was approximately .48 for both sexes.

In line with the suggestion from several authors [60,61], some findings indicate that these interpretations should be considered as complementary rather than mutually exclusive. For example, Gruenewald *et al*. specifically sought to examine how SEP at different time points from childhood to adulthood predicted AL, and found that SEP both cumulatively and at each time point independently predicted adult AL [11]. Previous findings have also indicated interactive effects, with early life factors increasing the individual’s vulnerability to later stress [18,62]. Further, considering the different types of exposure in the present study, it is likely that the validity of these interpretations differ across exposures. For example, Friedman *et al*. examined the differential influence of various types of self-reported early life exposures and found that childhood socioeconomic adversity, but not physical abuse, was mediated by adult education [16]. Thus, the present findings seem to require interpretations that allow for interplay between latent and pathway effects.

### Strengths and limitations

This study contributes to the existing literature on the association of childhood parental SEP with later life health by examining the association of specific early life factors that have previously been related to both parental SEP and adult health with midlife allostatic load. The observational, prospective data on both biomedical and social variables from a large population-based sample, and the multifaceted measure of parental SEP are among the primary strengths of the study. Additionally, the use of a multidimensional, preclinical outcome measure in a midlife population allows insight to antecedent mechanisms of commonly studied outcomes such as cardiovascular disease and mortality. Finally, the study addresses an important gap in the current literature by focusing on potential sex differences. However, certain limitations should be mentioned.

First, as referenced above, there are examples of studies reporting similar findings for men and women in the association of early life factors with adult AL [26], and a point of reservation regarding sex differences in this study is in order. In analyses of the full sample, formal tests of sex differences in the effects of early life factors were significant or marginally significant for four factors only (maternal smoking in the final trimester, complications at birth, maternal BMI and not living with parents at one year). That is, it is possible that there are no sex differences in most of these associations, and that the fewer significant findings for men is due to a smaller proportion of men in the sample. Resolving this issue requires further research. Also related to the study sample, a comparison of CAMB study participants with non-participants showed that participants were slightly better educated and that all-cause mortality registered from the beginning of the study to December 2012 was higher in non-participants [37], indicating the possibility of selection bias. Similarly, among members of the CPC sample who were invited to participate in CAMB, participants and non-participants were differentially exposed to several of the early life predictors: among the participants, fewer were exposed to maternal smoking or hospitalization within the first year, fewer were born to unmarried mothers and were characterized as unwanted by their mother, they were breastfed for a longer period of time and were born to older mothers and parents of higher parental SEP compared to those not participating in CAMB. This may have introduced a bias leading to an underestimation of effects, while the narrow age range of 49–52 years limits the generalizability the present findings.

Additional limitations relate to the AL measure. First, since there is currently no consensus as to how AL is best measured, the comparability of the present findings to those of other studies using alternative methods of assessment may vary [63]. Sensitivity analyses showed slightly higher AL-scores when derived from clinical cut-points, reflecting increased levels of dysregulation which is to be expected in a midlife study population. Using this measure induced slight changes in the effects of some predictors. While sample-derived cut-off-scores continue to be most widely used, this finding highlights the importance of evaluating different operationalization techniques with the aim of increasing the comparability of findings related to AL across studies [64]. Further, within the AL framework, neuroendocrine biomarkers such as cortisol are considered primary mediators [55]. As there were no neuroendocrine biomarkers available for the present study sample, these findings relate only to secondary outcomes, hypothesized to be related to dysregulated HPA axis and sympathetic nervous system activity.

## Conclusion

This is one of the first prospective studies on early life predictors of midlife AL. Parental SEP and maternal BMI significantly predicted AL in both sexes, though the latter association was found to be stronger in men. In men, additional significant associations were found for attitude towards the pregnancy, whereas complications at birth, birth weight and not living with parents at one year significantly predicted AL in women. While the study contributes information on the effects early life factors which have not previously been examined in relation to AL, the findings confirm the previously established predictive validity of parental SEP on adult allostatic load, as this association was only slightly attenuated when adjusted for other early life factors known to be associated with SEP and health. Finally, the results suggest a need for further investigation of sex differences in the associations of early life factors with AL.

## Supporting information

S1 Table Biomarker cut-points. Sex-stratified means or medians and risk cut-points for 1,718 participants with blood samples.
(DOCX)

S2 Table Zero-order correlation matrix of main study variables. (DOCX)
