# Correction: Early life predictors of midlife allostatic load: A prospective cohort study

**DOI:** 10.1371/journal.pone.0222732

**Published:** 2019-09-12

**Authors:** Dinne Skjærlund Christensen, Trine Flensborg-Madsen, Ellen Garde, Åse Marie Hansen, Jolene Masters Pedersen, Erik Lykke Mortensen

There are errors in Table 1. The value in Women, Biomedical factor, “Maternal smoking in the final trimester (N[%])” should be 436. The value in Women, Social factor, “Attitude toward the pregnancy, unwanted (N[%])” should be 472.

**Table 1:** Sex-stratified descriptive statistics of early life biomedical and social factors including test for sex differences.

	n	Men	n	Women	*p*^a^
Age at follow-up (mean[min; max])	726	49.9 (49;52)	922	49.9 (49;52)	.57
Allostatic load score (mean[SD])	726	3.47 (2.66)	922	3.42 (2.74)	.72
Biomedical factors					
Maternal smoking in the final trimester (N[%])	710	328 (46.2)	899	436 (48.5)	.36
Gestational age, weeks (mean[SD])	591	39.07 (2.6)	778	39.2 (2.3)	.24
Complications at birth (N[%])	711	66 (9.3)	904	71 (7.9)	.31
Hospital stay in the first year (N[%])	712	137 (19.2)	892	146 (16.4)	.13
Birth weight, grams (mean[SD])	718	3326 (587.5)	906	3178 (540.5)	< .001
Maternal BMI (mean[SD])	659	21.7 (2.85)	839	21.6 (2.81)	.29
Duration of breastfeeding, months (mean[SD])	625	3.3 (2.76)	785	3.3 (2.70)	.99
Maternal age at birth, years (mean[SD])	726	26.2 (6.66)	922	25.9 (6.71)	.47
Social factors					
Attitude toward the pregnancy, unwanted (N[%])	704	351 (49.7)	889	472 (53.1)	.20
Marital status of mother at conception, single (N[%])	716	211 (29.5)	910	303 (33.3)	.099
Change in marital status, conception to 1 year (N[%])	654	76 (11.6)	821	108 (13.2)	.38
Not living with parents at 1 year (N[%])	726	34 (4.7)	922	33 (3.6)	.26
Parental SEP at 1 year, 1–8, low to high (mean[SD])	610	4.38 (1.88)	777	4.33 (1.89)	.62

^a^ *t*-test or *x*^2^ test for sex differences
