# Supplementary material for: Early life predictors of midlife allostatic load: A prospective cohort study
Source: PLoS One. 2018 Aug 16;13(8):e0202395. doi: 10.1371/journal.pone.0202395 (PMC6095582; doi:10.1371/journal.pone.0202395)
Supplement: S1 Table — Sex-stratified means or medians and risk cut-points for 1,718 participants with blood samples. (DOCX) [file pone.0202395.s001.docx]

**S1Table. Biomarker cut-points.** Sex-stratified means, medians and risk cut-points for 1,718 participants with blood samples.

|  | **Men** | | | | |  |  |  | **Women** |  |  |  |  |
| --- | --- | --- | --- | --- | --- | --- | --- | --- | --- | --- | --- | --- | --- |
| **Biomarker** | **n** | **Mean** | **Median** | **SD** | **Cut-point** |  | **n** | **Mean** | **Median** | **SD** | **Cut-point** |  | **Clinical cut-point** |
| Diastolic blood pressure (mmHg) | 752 | 88.5 | 87.3 | 10.3 | > 94.75 |  | 965 | 84.4 | 83.8 | 10.6 | > 90.5 |  | > 90 |
| Systolic blood pressure (mmHg) | 752 | 135.7 | 134.8 | 14.9 | > 143.5 |  | 965 | 124.8 | 122.3 | 16.5 | > 133 |  | > 140 |
| Body Mass Index (kg/(m)^2^) | 752 | 26.7 | 26.04 | 4.12 | > 28.9 |  | 966 | 25.5 | 24.63 | 5.05 | > 27.7 |  | > 30 |
| Waist/Hip ratio | 752 | 0.94 | 0.94 | 0.06 | > 0.97 |  | 965 | 0.85 | 0.85 | 0.06 | > 0.89 |  | - |
| Body fat (%) | 752 | 21.1 | 20.9 | 6.07 | > 25.2 |  | 962 | 31.1 | 31.3 | 7.00 | > 35.8 |  | - |
| Blood glucose (mmol/l) | 748 | 5.43 | 5.20 | 1.43 | > 5.80 |  | 958 | 5.27 | 5.20 | 1.05 | > 5.70 |  | > 7.0 |
| HbA1c (% of total hemoglobin) | 749 | 5.25 | 5.22 | 0.55 | > 5.50 |  | 950 | 5.09 | 5.08 | 0.47 | > 5.35 |  | > 7.0 |
| Total cholesterol (mmol/l) | 749 | 6.27 | 6.21 | 1.20 | > 7.01 |  | 950 | 6.13 | 6.06 | 1.09 | > 6.86 |  | > 5.0 |
| High-density lipoprotein (mmol/l) | 749 | 1.37 | 1.35 | 0.34 | < 1.12 |  | 950 | 1.68 | 1.64 | 0.40 | < 1.41 |  | < 1.20; 1.0^a^ |
| Low-density lipoprotein (mmol/l) | 749 | 3.30 | 3.22 | 0.89 | > 3.84 |  | 950 | 2.99 | 2.94 | 0.85 | > 3.52 |  | > 3.0 |
| Triglycerides (mmol/l) | 749 | 2.09 | 1.76 | 1.30 | > 2.49 |  | 950 | 1.46 | 1.27 | 0.76 | > 1.77 |  | > 1.70 |
| hsCRP (mg/ml) | 747 | 2.29 | 1.20 | 4.62 | > 2.30 |  | 948 | 2.39 | 1.10 | 4.99 | > 2.40 |  | - |
| Interleukin-6 (pg/ml) | 743 | 2.42 | 1.52 | 3.98 | > 2.39 |  | 946 | 2.93 | 1.50 | 15.0 | > 2.45 |  | - |
| TNF apha (pg/ml) | 745 | 5.60 | 4.47 | 9.34 | > 5.31 |  | 946 | 5.00 | 4.06 | 6.95 | > 4.96 |  | - |

^a^Sex-specific cut-points: women; men
