# Supplementary material for: Early life predictors of midlife allostatic load: A prospective cohort study
Source: PLoS One. 2018 Aug 16;13(8):e0202395. doi: 10.1371/journal.pone.0202395 (PMC6095582; doi:10.1371/journal.pone.0202395)
Supplement: S2 Table — (DOCX) [file pone.0202395.s002.docx]

**S2 Table**. Zero-order correlation matrix of main study variables.

|  | 1 | 2 | 3 | 4 | 5 | 6 | 7 | 8 | 9 | 10 | 11 | 12 | 13 | 14 |
| --- | --- | --- | --- | --- | --- | --- | --- | --- | --- | --- | --- | --- | --- | --- |
| 1. Allostatic load | 1 |  |  |  |  |  |  |  |  |  |  |  |  |  |
| 2. Maternal smoking | 0.06* | 1 |  |  |  |  |  |  |  |  |  |  |  |  |
| 3. Gestational age | 0.04 | -0.02 | 1 |  |  |  |  |  |  |  |  |  |  |  |
| 4. Complications at birth | 0.03 | -0.01 | -0.04 | 1 |  |  |  |  |  |  |  |  |  |  |
| 5. Hospital stay in the first year | 0.04^†^ | 0.11*** | -0.29*** | 0.06* | 1 |  |  |  |  |  |  |  |  |  |
| 6. Birth weight | -0.05* | -0.22*** | 0.52*** | -0.01 | -0.33*** | 1 |  |  |  |  |  |  |  |  |
| 7. Maternal BMI | 0.13*** | -0.07** | 0.06* | 0.02 | -0.01 | 0.14*** | 1 |  |  |  |  |  |  |  |
| 8. Duration of breastfeeding | -0.06* | -0.09** | 0.01 | 0.03 | -0.15*** | 0.06* | -0.05^†^ | 1 |  |  |  |  |  |  |
| 9. Maternal age at birth | -0.08** | -0.05* | -0.11*** | 0.02 | -0.09*** | 0.05^†^ | 0.22*** | 0.10*** | 1 |  |  |  |  |  |
| 10. Attitude toward the pregnancy | 0.04^†^ | 0.06* | 0.04 | -0.01 | 0.07* | -0.02 | 0.04 | -0.16*** | -0.28*** | 1 |  |  |  |  |
| 11. Marital status of mothers at conception | 0.10*** | 0.12*** | 0.08** | -0.03 | 0.06* | -0.08** | -0.04 | -0.18*** | -0.39*** | 0.45*** | 1 |  |  |  |
| 12. Change in marital status, conception to 1 year | 0.09** | 0.01 | 0.04 | -0.02 | 0.00 | -0.01 | -0.07* | -0.09** | -0.24*** | 0.24*** | 0.50*** | 1 |  |  |
| 13. Not living with parents at 1 year | 0.04 | 0.09*** | 0.01 | -0.03 | 0.07** | -0.02 | 0.02 | -0.18*** | -0.09*** | 0.18*** | 0.23*** | -0.00 | 1 |  |
| 14. Parental SEP at 1 year | -0.20*** | -0.13*** | -0.04 | 0.00 | -0.14*** | 0.06* | -0.07* | 0.17*** | 0.40*** | -0.36*** | -0.38*** | -0.26*** | 0.03 | 1 |

† *p* < .10; * *p <* .05; ** *p <* .01; *** *p <* .001
